# *Epilepsia Open*: Welcome to the inaugural lived experience editor and editorial interns

**DOI:** 10.1002/epi4.12860

**Published:** 2023-11-10

**Authors:** Aristea S. Galanopoulou

**Affiliations:** ^1^ Saul R. Korey Department of Neurology, Isabelle Rapin Division of Child Neurology, Dominick P. Purpura Department of Neuroscience Albert Einstein College of Medicine, Montefiore/Einstein Epilepsy Center Bronx New York USA

We are excited to welcome and introduce the inaugural lived experience editor and editorial interns among our editorial board. Being a fully open‐access journal, we believe that improving the way we communicate the advances or epilepsy research and care to the public is vital for conveying a transparent and accurate message to the community. Furthermore, by opening up the editorial process to a younger generation investigators, through mentored editorial internships, we hope to provide a valuable experience for career advancement by engaging them in a vital function of research.

With these in mind, we recently opened a call for a “lived experience editor” to join us and assist with the implementation of changes in the manuscript format and content of the journal. After reviewing the applications we received, we are delighted to welcome Action Amos as our inaugural lived experience editor (Figure 1). Action has been committed to incorporating the lived experience perspective in healthcare research and care for people with epilepsy and in highlighting gaps and improvements in these areas. He received an MBA in leadership and sustainability from Cumbria University, an MSc in mental health in children and young people from the University of Edinburgh, and is currently pursuing his PhD at the same University. His research has centered around ethical aspects of research, involvement of users and caregivers in decision‐making processes in low‐resource countries, engagement of people with disabilities in research, and neurocysticercosis. Action has served as vice president of the International Bureau of Epilepsy, Africa (2018–23) and member of task forces of the International League Against Epilepsy (ILAE) and has been involved in creating a roadmap for implementation of the World Health Organization intersectoral global action plan for epilepsy as a target. We are looking forward to working with Action in bringing research published in *Epilepsia Open* closer to the community and also in rendering the community's voice stronger within the epilepsy research community. The first step in this effort is the implementation of plain language summaries in all our published articles and the encouragement of graphical abstracts conveying important concepts and results.

**FIGURE 1:**
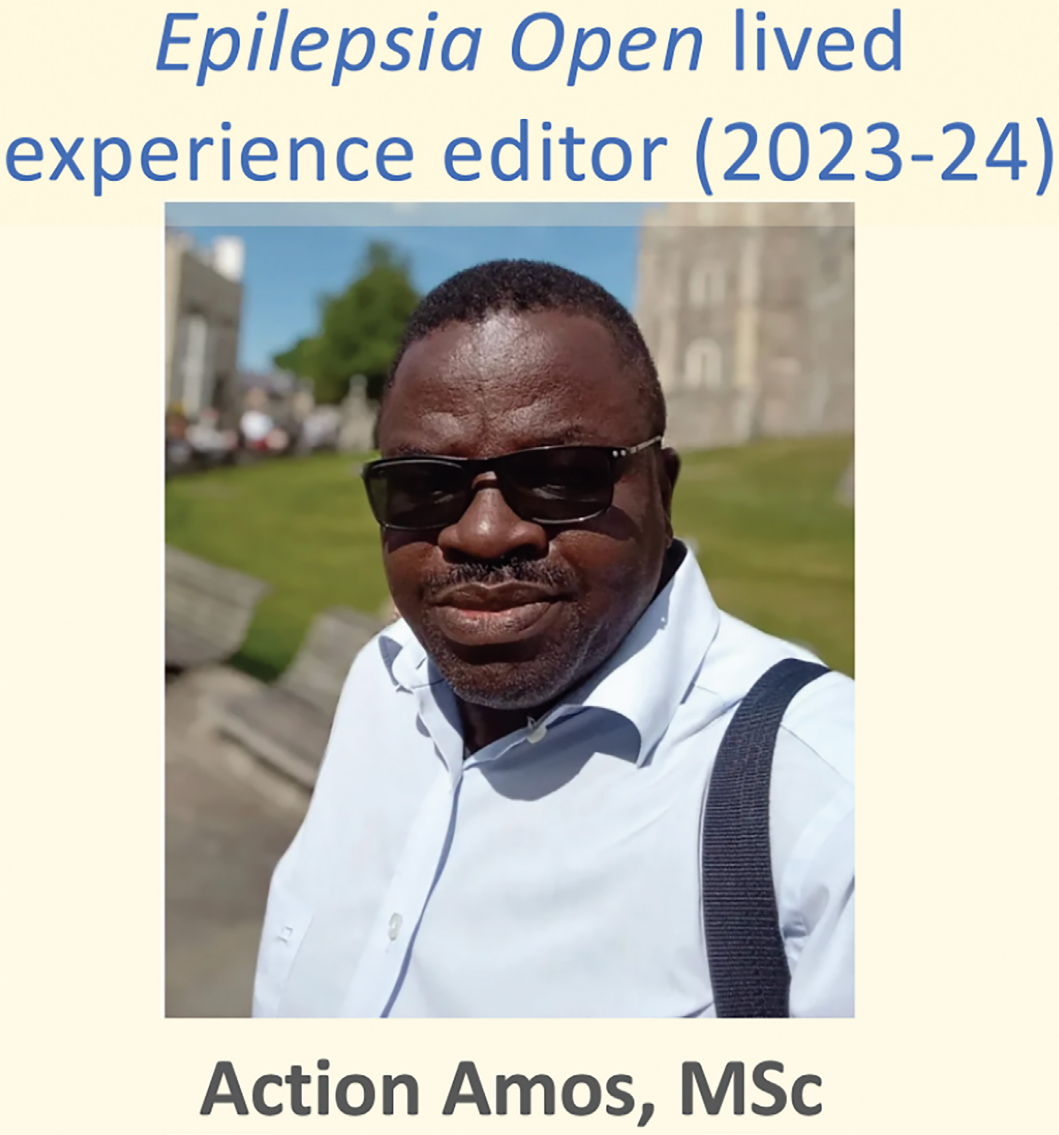
Action Amos, MSc, lived experience editor of *Epilepsia Open* (2023–24).

In response to a call for internships to our editorial board, we received many applications from talented investigators across the globe. The editorial board, following an internal voting process, selected four inaugural editorial interns for 2023–24 (Figure 2), who have diverse research interests and expertise.

**FIGURE 2:**
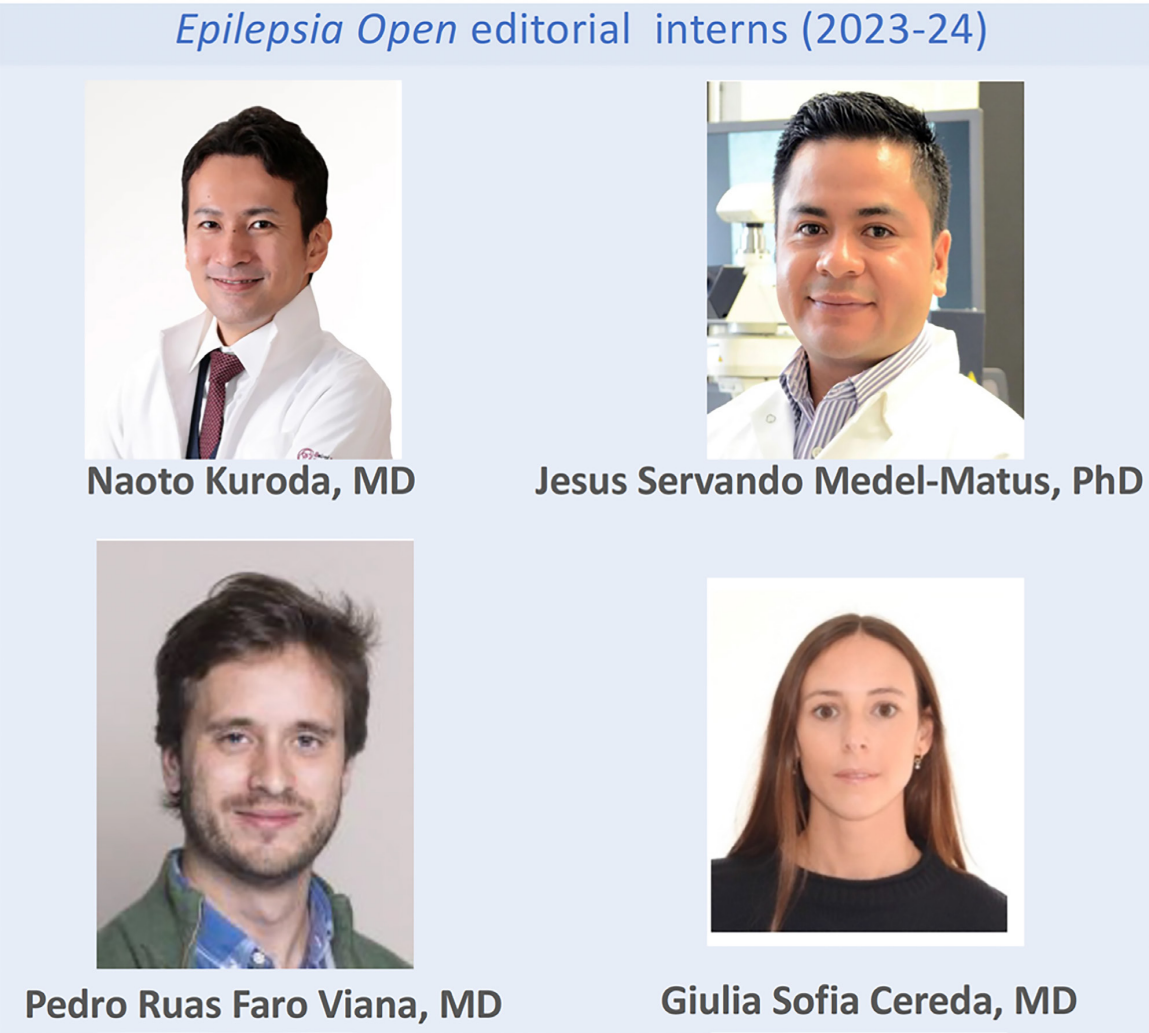
Inaugural editorial interns of *Epilepsia Open* (2023–24).

Naoto Kuroda received his MD at the Jikei University School of Medicine (Japan), is a board‐certified neurosurgeon, currently enrolled in PhD studies at Tohoku University, and a research fellow at Wayne State University (Detroit, MI, USA). He has been very active within the ILAE, ILAE Young Epilepsy Section (YES), has received several awards, and has special interest in electrophysiological biomarkers for epilepsy surgery outcomes, epilepsy surgery, and the impact of the COVID‐19 pandemic.

Jesus Servando Medel Matus received his PhD at the Institute of Neuroethology at the University of Veracruz, Mexico, did postdoctoral research at UCLA, and is currently an associate project scientist at the developmental epilepsy research laboratories at the David Geffen School of Medicine in Los Angeles. He has numerous publications on models of epilepsy, the role of neuroinflammation and neuropeptides in seizure control and epilepsy‐related behavioral comorbidities, as well as on the role of gut microbiome in kindling and post‐traumatic epilepsy. He was the recipient of the *Epilepsia Open* Basic Science Prize in 2019.

Pedro Viana obtained his medical degree (NOVA Medical School) and neurology training (Centro Hospitalar Lisboa Norte) in Portugal, completed a clinical scholars research training program through Harvard Medical School and Fundação para a Ciência e Tecnologia, and is a PhD candidate in neuroscience (University of Lisbon). He is currently a clinical research fellow and locum consultant neurologist at King's College (London, UK). His research is on seizure forecasting and reporting, chronobiology, and clinical neurophysiology.

Giulia Sofia Cereda received her MD at the University of Genova (Italy) and is currently a neurology resident at the University of Milano‐Bicocca (Milan, Italy). She is training in clinical epileptology at the Epilepsy Unit of the IRCCS Foundation Carlo Besta Neurological Institute and at the Epilepsy Center of the IRCCS San Gerardo dei Tintori (Italy), participating in several research projects within the field of epilepsy. Her main interests include immune‐mediated epilepsy and epilepsy surgery.

The internship will allow these talented young investigators to experience, first hand, the peer review and editorial system, and obtain mentorship by outstanding experts of the editorial board of *Epilepsia Open* with diverse research interests. We welcome the new additions to our editorial board and are looking forward to their contributions toward the growth of the journal.

## CONFLICT OF INTEREST STATEMENT

None of the authors has any conflict of interest to disclose. I confirm that I have read the Journal's position on issues involved in ethical publication and affirm that this editorial is consistent with those guidelines.

